# Around the Hospitals

**Published:** 1892-11-12

**Authors:** 


### AROUND THE HOSPITALS.

**The Longmore Hospital for Incurables** is the only institution of its kind for the south-east of Scotland. During the past two years no less than 209 applications have been received for admission, the institution possessing but 50 beds. Additional space is, however, about to be added, and a new wing for 34 patients is in the course of completion. This will necessitate the extension of both kitchen and laundry. It is expected that this extension, with partial endowment, will cost £15,000. Of this, £10,237 is already secure from various sources, but an appeal for nearly £5,000 is necessary. Besides the general accommodation, the institution possesses four rooms, capable of receiving two persons in each, for paying patients. These were especially provided at the request of Mr. Longmore, that the wants of his own class might be considered. Paying patients are received at the rate of £52 per annum, or, if sharing a room, at £35. Patients of all nationalities are received, and therefore the claims of the Longmore Hospital extend beyond the limit of Scotland. During the past year the annual subscriptions have shown a falling off, but this, it is stated, arose largely from the fact that many collectors were incapacitated by influenza, and we trust that the deficiency will be made up during the present year. The class of patients received in the incurable hospital is one that should especially appeal to our generosity.

